# Paris

**Published:** 1894-07-07

**Authors:** 


### Continental Notes from a Holiday Correspondent.

PARIS—(*continued*).

Besides about twelve general hospitals, there are a large number of special hospitals in Paris directly under State control, and most of these are open to visitors. Two only I shall notice—the Hospital Broca and the Quinze-Vingts—both special hospitals. The former is in the rue Broca, rather a long way out on the south side of the Seine. It is a women's hospital, and here M. Pozzi, the famous gynæcologist, is to be seen; he is surgeon to the institution. M. Pozzi is quite a young man; he speaks English, and makes English visitors very welcome. He has about seventy beds, and attends daily at ten o'clock, doing major operations almost every day. He is rigidly aseptic, and the great feature of his operations is their conservatism, only removing diseased organs or portions of organs. He is the introducer of ignipuncture of the ovaries and resection of portions of ovaries. His work includes operations on all the abdominal organs, and his results are extremely good.

The Quinze-Vingts is in the rue de Charenton, three minutes' walk from the Place de la Bastille. It was founded in 1260 by St. Louis for the accommodation of three hundred poor blind persons. A clinic and ophthalmic hospital are now annexed, and an enormous amount of work is done. There are four surgeons and a director of the laboratory. M. Trousseau, whom I saw, operates on Friday at one o'clock, and his operations are well worth going to see. An American told me before going that "It was jugglery, not surgery." But after seeing for myself, I think it is surgery of a very high order. M. Trousseau does extraction of cataract with absolutely no instruments but a Graefe's knife and a spatula. By a slight turn of his hand he divides the capsule while making the incision in the cornea, and then extracts the lens without iridectomy. All the while he keeps the lid open and the eye fixed with the fingers of his left hand. I timed him several times, and found that from the time he touched the eye till the lens was extracted was less than thirty seconds, and in less than two minutes the wound was dressed, the eye bandaged, and the patient ready to be removed, yet there was no sign of hurry. From his report I find that in 1893 he did 194 extractions of senile cataract, of which 175 were simple extractions. In these there was one case of suppuration of the wound, one of escape of vitreous, and one of panophthalmia. The total number of operations (not including minor ones) done by all four surgeons last year was 1,957, and of these 822 were for cataract. More than 16,000 new patients were seen during the year.

We had fully intended to visit the Pasteur Institute, but it is in a rather out-of-the-way part of Paris, and visitors are only admitted at half-past eleven in the morning, an hour which did not fall in with our other arrangements. It has so often been described, however, that we did not much mind missing it. Even the most energetic medical man will have a good deal of spare time on his hands each day after visiting the clinics, and some hints as to how this may be spent will be useful. The clinics are always hot and often noisy, and after some hours spent at them, the best preparation for the next day's work is to avoid the hot, and crowded places of amusement in the city and go off to the country for a few hours. The environs of Paris though not, perhaps, so beautiful as those of London, are still exceedingly attractive, especially the extensive woods which almost surround the city at some distance.

Versailles and St. Germain are the two places most frequented by excursionists, and described in every guide book. The greater part of the day is required for either. Of the places nearer Paris, none is more lovely than St. Cloud, which is best reached by the Seine steamers. These run every few minutes from the Port Royal, opposite the Louvre, and take one an hour's run down the river for the very moderate sum of twenty centimes. They stop at numerous places on the way, ending at Suresnes. St. Cloud is near the end of the run, on the left bank. There is a small village, but one turns to the left, away from it, into the gate of the park. The palace which used to be here was ruined in the war of 70-71, but all traces of it have now been removed, and its site covered with terraces and flower beds. Keeping up the hill, past the fish-ponds, one gets a glorious view of Paris and the Seine, which is worth going a long way to see. After a rest at the top o the hill, one may wander for miles through the woods, which extend several miles back from the river, or one can walk along the heights above the river, keeping parallel to it, and come out at Sevres, a clean, picturesque little town, the seat of the famous porcelain works.

Another day one may go down the river by boat to Suresnes, and cross the bridge there into the Bois de Boulogne, either returning the same way or walking across the Bois to the gates on the town side, where trams and buses are to be found.

A very interesting excursion may be made up the river, instead of down, in the boat to Charenton, a little town at the junction of the Seine and Marne. These boats keep to the left bank of the river; a stopping place will be found just by the Port Royal. At Charenton one may leave the river and wander through the Bois de Vincennes, see the fine old fortress at Vincennes, and take the tram home from there; or one may walk by the towing-path up the Marne, and return the same way to the boat.

